# Effect of regionalized structures on rock fracture process

**DOI:** 10.1038/s41598-024-60849-2

**Published:** 2024-05-07

**Authors:** Xulong Yao, Zhen Liu, Yanbo Zhang, Zhigang Tao, Peng Liang, Jizhong Zhao

**Affiliations:** 1 College of Mining Engineering, North China University of Science and Technology, Tangshan, 063210 China; 2 Hebei Provincial Laboratory of Mining Industry Development With Safe Technology Priority, Tangshan, 063210 China; 3 Hebei Green Intelligent Mining Technology Innovation Center, Tangshan, 063210 China; 4 State Key Laboratory for Geomechanics & Deep Underground Engineering (China University of Mining & Technology), Beijing, 100083 China; 5 School of Mechanics and Civil Engineering, China University of Mining & Technology, Beijing, 100083 China; 6 Shougang Luannan Macheng Mining Co., Ltd, Tangshan, 063501 China

**Keywords:** Engineering, Materials science

## Abstract

The structure of rocks plays a crucial role in their failure process. However, it is ignored that the interactions between rock internal structure and the effect of its own evolution on the rock fracture process. To investigate the effect between the evolution law of rock regionalized structures and their interaction relationships during failure. We conducted an experiment using visual acoustic imaging monitoring to study rock failure, introducing a new concept of characteristics of rock structure—regionalized structures. The findings reveal three main types of regionalized structures in rocks: skeleton regions, variable regions, and damage regions. These structures combine to form four categories of complex rock structures: block-type support skeletons, point column-type support skeletons, suspension-type weak support skeletons, and no skeletons. During the failure process, we found that these regionalized structures worked together synergistically to control rock failure. Although the evolutionary relationships among the structures show some similarities, the final fracture states vary significantly. Stress and strain distribution patterns clearly demonstrate that variations in the force capacities and roles of the regionalized structures influence the synergistic evolutionary relationships, ultimately impacting the mode of rock failure. This work provides new insights for further research on rock failure mechanisms and can significantly contribute to preventing rock engineering disasters related to regionalized structures.

## Introduction

The impact of rock structure on rock failure is a key focus in the field of rock mechanics^1–4^. Rocks are complex geological formations with various defects such as cracks, holes, and structural surfaces. These defects contribute to differences in the rock’s ability to withstand forces and in the process of damage evolution^5–8^. Discovering the evolutionary relationships between rock structure and failure damage has great importance in the field of rock mechanics and engineering.

In recent years, numerous research studies have focused on studying the influence of microstructure on fracture processes in rocks^9–12^. However, due to the complexity of microstructure and the interdependent nature of microstructures, understanding their impact on rock failure remains a challenge. Scholars have encountered difficulties in establishing a clear relationship between the evolution of microstructure groups and rock failure. It is important to note that microstructures rarely exist in isolation, but rather interact and combine to affect rock failure. This interaction arises from the joint action of rock forming and storage environments, often guided by spatial regional correlation. Therefore, conducting an indepth study on the relationships between regionalized structure and rock failure can provide fresh insights into the mechanisms of rock failure and make a significant contribution to mitigating rock engineering problems associated with regionalized structures.

Numerous scholars have conducted extensive studies on the relationship between rock structure and its macromechanical properties^13–17^. These studies use the mechanical properties of rocks to evaluate stability and predict disasters^18–20^. The findings consistently demonstrate that rock structure significantly influences the mechanical properties. For example, Zhang et al.^21^ used computed tomography (CT) scan tests to investigate shale's mechanical properties based on macrostructure and hydration, revealing differences due to variations in structure. Li et al. ^22^ propose a micromechanical damage model based on various experiments and artificially reconstruct the microstructure of the studied rocks using Fast Fourier transforms (FFT). Comparisons between experimental data and numerical predictions showed that the proposed model is well able to predict the macroscopic mechanical behavior of the rocks, and the peak stress and post peak behavior of the rock are strongly related to the rock structure damage localization, and the structure characteristics indeed greatly influence the mechanical behavior. Huang et al.^23^ analyzed rock structure, homogeneity, and mechanical properties using Scanning electron microscope (SEM), finding a correlation with macroscopic mechanics. Ersoy et al.^24^ quantitatively measured the main structural features of rocks using an IBAS 2000 automatic image analysis system (such as grain size, shape, and orientation). The structural coefficients were used to represent the structural characteristics of each specimen., and their relationship with the rock's mechanical properties was statistically established. The result demonstrated that the rock structure can be used as a predictive factor for mechanical. Torquato et al. ^25^ presented several idealized models of the microstructure, explained the statistical characterization of the microstructure of these materials, and confirmed that changes in rock structure significantly affect mechanical properties based on mathematical analysis. In summary, through their research, these scholars collectively show that rock structure plays a major role in determining its macroscopic mechanical properties.

However, many present rock structure studies only rely on very overview parameters, such as porosity, homogeneity coefficient, and so on. It is undeniable that these parameters do promote the development of the field of rock mechanics. But in fact, the study disregards structural regional properties and structural interactions. This study highlights the significant impact of synergistic evolution on rock failure.

Over the past few decades, scholars have extensively studied the impact of rock structure on failure processes. Yu et al. ^26^ obtained the distribution of rock structure based digital image processing (DIP) and imported it into rock failure process analysis (RFPA) in order to examine the effect of rock structure on the rock failure process. The numerical results indicate that the rock structure characteristics may further control the failure process. Liu et al.^27^ conducted triaxial tests on gneisses, finding that structure and stress conditions strongly affect strength and failure. Xu et al.^28^ built numerical models reflecting coal's micro-structure characteristics, revealing that different structures greatly influence failure. Wu et al.^29^ proposed an image-based finite-discrete element method (FDEM) model to investigate nonlinear mechanical behaviour and failure mechanisms. They demonstrated that rock microstructures affect failure and mechanical behaviour. Li et al.^30^ proposed a multilevel parallel bonded-grain model to reveal the influence of the microstructure characteristics of granite. The comprehensive simulations are conducted to examine the dynamic damage evolution of brittle granitic rock, and the behaviors of the microfractures are discussed based on fish function. Song et al.^31^ comparatively analyzed based on the perspectives of energy evolution, rock burst starting, and mesoscopic characterization and believe that the research view of the progressive failure process and fracture mechanism of rocks under compression should be transferred. Therefore, the structural evolution theory to reveal the progressive failure process and fracture mechanism of rocks is proposed, and the evolution characteristics of the four stages and progressive failure mechanism of rocks under compression are better verified. Zhang et al.^32^ constructed a digital model of rock based on a polarizing microscope, computed tomography (CT), and digital image processing, which can better represent the fracture process of the rock internal structure and better reveal the rock fracture mechanism from the level of the mineral structure. Fedotov et al.^33^ propose an evolution model of rocks under mechanical stress based on the kinetic approach, and indicate that rocks can be considered as self-similar hierarchical structures based on experimentally verified criteria for material destruction, to trace the evolution of rocks under mechanical stress from an ideal defect-free state to complete destruction. The above scholars examined rock failure from multiple perspectives, showing the fundamental role of rock structure evolution.

Our research recognizes a notable problem in studying the evolutionary process of rock failure. It offers a limited scope that only considers relevant mechanical curves, failing to capture internal structure information and explore synergistic evolutionary relationships between structural masses. Rocks are naturally composite and heterogeneous materials, with self-mechanic differences resulting in structural variations. Perhaps, the synergistic evolution of rock structures during failure is a reason for the irreproducibility of rock mechanics experiments. Research on the synergistic evolutionary relationships between regionalized structures under external forces is necessary for new insights into rock failure. Meanwhile, the whole failure process of rocks needs to be detailed and characterized with more intuitive views, which can better contribute to the problem of visualizing rock fracture.

In recent years, methods of internal visualization of the rock have been gradually developed to reveal the evolution law of rock structure and the dynamic evolution information of the rock in order to solve the "black box" problem in the rock fracture process. Among these methods, acoustic imaging technology can detect the evolution of the internal structure during the rock fracture process, and as an effective detection means, it has been extensively applied to experimental mechanics and other fields. This method is mainly based on the AST (auto sensor testing) function of acoustic emission monitoring equipment to construct a dynamic damage detection imaging method based on the correlation of the area inside the rock, which can better characterize the internal structure of the rock^34^. Zhang et al.^35^ conducted the uniaxial multi-stage loading test based on acoustic imaging technology and analyzed mechanical behavior during rock failure. The results show that the wave velocity imaging have a great effect on the extent and spatial position of damage to the rock with three-dimensional (3D) visualization, and based on acoustic emission (AE) parameters and wave velocity imaging, describe the changes in activity of cracks during rock fracture from temporal and spatial perspectives. Wang et al.^36^ carried out a multi-stage loading test to obtain the imaging results with acoustic imaging technology, analyze the wave velocity evolution characteristics of the four stages in the rock fracture process, and quantitatively describe the evolution characteristics of the wave velocity field.

In summary, it is now widely believed that rock structures exist in isolation, and the study of rock structure is such an such an overview that it not only ignores the regional properties of the internal structure of rock but also the interactions between rock internal structure and the effect of its own evolution on the rock fracture process. In view of this, we conducted a uniaxial multistage loading test using acoustic emission-acoustic imaging monitoring. Meanwhile, we analyzed the imaging results to define and analyze the distribution relationships of regionalized structures in rocks. Additionally, we investigated the synergistic evolutionary rules during rock failure. This research provides new ideas for studying rock failure mechanisms, and the conclusion obtained would better apply to and can offer effective early warning for potential damage in engineering projects involving regionalized structures, like slopes and tunnels surrounding rock.

## Materials and methods

### Specimen preparation

The siltstone with better structural characteristics was selected for testing. The siltstone specimens used in this study were obtained from Laizhou City, Yantai, Shandong Province, China. Drilling cores without obvious fractures were selected and cut into cubes with dimensions of approximately 100 × 100 × 100 mm to meet requirements for routine mechanical tests on rocks. The uniaxial compressive strength is 67.917 MPa, the elastic modulus is 6.182 GPa, the poisson ratio is 0.3, and the density is 2.6 g/cm^3^. There are twelve cube specimens for the experiment, numbered from FS-1 to FS-12. Each rock sample meets the ‘International Society for Rock Mechanics (ISRM)'. Both sections of each rock sample are flat and smooth, and the ends of the specimen are not more flat than ± 0.01 mm, and both ends of the sample are perpendicular to the axis of the sample.

### Testing equipment and settings

**Figure 1:**
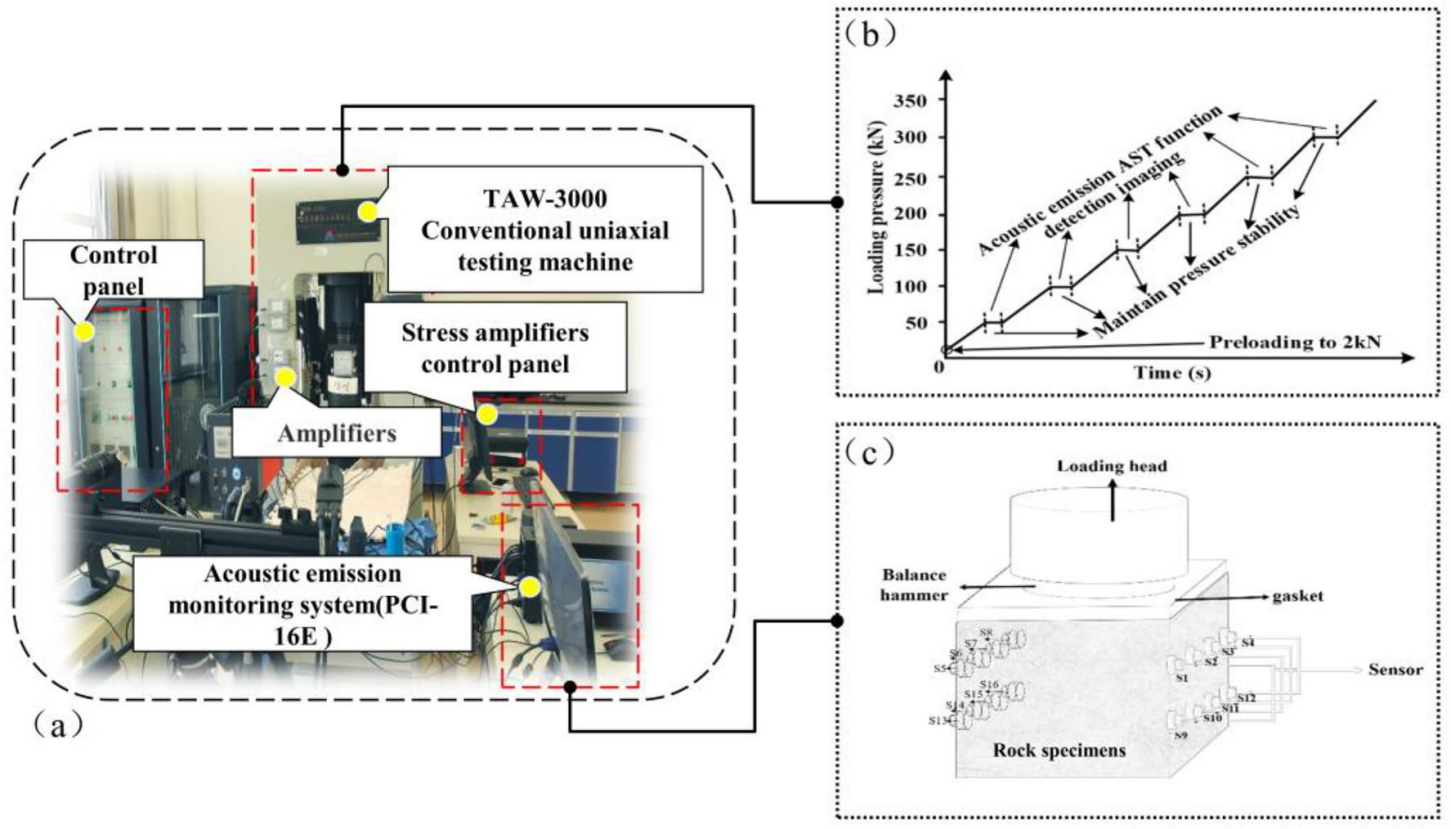
Schematic diagram of test device and sensor layout. (**a**) laboratory test loading system (**b**) Schematic diagram of mechanical loading path (**c**) Schematic diagram of sensors layout.

The testing was conducted using a TAW-3000 testing system, capable of uniaxial compression tests with monitoring methods like acoustic emission (AE), infrared thermal imaging, and full field strain (Fig. 1a). The system has a maximum axial capacity of 3000 kN, an effective measurement range of 30–3000 kN, and a maximum lateral confining pressure of 1000 kN. The displacement control loading speed ranges from 0.005 to 100 mm/min, and it can operate continuously for 5000 h.

For this testing, the axial displacement was controlled using the uniaxial loading method at a rate of 0.2 mm/min, meeting rock mechanics experimental requirements and the mechanical loading path as shown in Fig. 1b. The monitoring system used a modified PCI-16E acoustic emission system with 16 R6α sensors arranged in an array. Vaseline coupling ensured proper contact between the sensors and the rock surface (Fig. 1c).

### Imaging method

**Figure 2:**
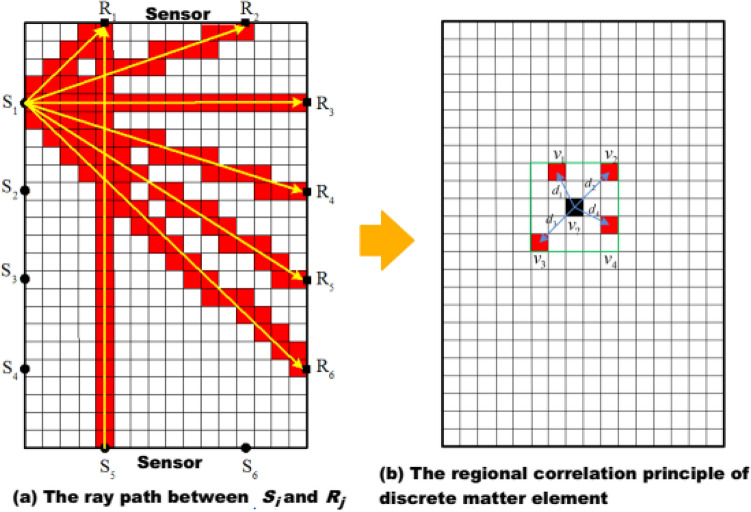
The imaging principle based on regional correlation.

The imaging method primarily employs the auto sensor test (AST) function of the acoustic emission monitoring system to sequentially excite the sensors to emit acoustic waves, collect the received signals from other sensors as well as the signal arrival time, and then calculate the approximate wave velocity on the linear path between the sensors (as shown in Fig. 2a). The wave velocities on the linear lines of different sensors are regarded as known information, and the valued wave velocities in the areas beyond the linear paths inside the rock are calculated using the kriging valuation method in geostatistics. Finally, the wave velocities in all sections of the rock are calculated to create a wave velocity field within the rock (as shown in Fig. 2b).

Assume that the rock is separated into several matter-elements and that each matter-element corresponds to a certain local area of the rock. The ray path is the composition of the matter-element passing through the straight line between *S*_*i*_ and *R*_*j*_ (such as red matter-element in Fig. 2a). The velocity of the red matter-element is the actual measured velocity between *S*_*i*_ and *R*_*j*_. The velocity of the white matter-element will be evaluated, and the initial value is zero.

When the size of a matter-element reaches a certain relative scale (such as mm or micron), the damage degree of the matter-element is related to its adjacent matter-element. At the same time, the wave velocity of a matter-element is related to its adjacent matter-element. As seen in Fig. 2b, the black cell is the matter-element whose velocity is unknown. The red cells are the matter-element whose velocity is known, and they are close to the black cell. It can be considered that the velocity of black cell is related to the velocity of red cells. Thus, the velocity of black cell is expressed as

1 $$ v_{?} = \lambda_{1} v_{1} + \lambda_{2} v_{2} + \lambda_{3} v_{3} + \lambda_{4} v_{4} $$

2 $$ \lambda {\text{i}} = \frac{{1/d_{i}^{p} }}{{\sum\nolimits_{i = 1}^{n} {(1/d_{i}^{p} )} }} $$

where λ_*i*_ (i = 1, 2, 3,…,n) is the weighting factor determined by the degree of correlation between the elements, and the calculation method can be referred to as Eq. (2). Where *d*_*i*_ (i = 1, 2, 3,…,n) is the straight line distance between the element whose wave speed is to be estimated and the ith neighboring element, and p is power. The initial wave velocity field of the rock before loading is denoted as *V*_*0*_, and the wave velocity field of the rock damage evolution process during loading *V*_*i*_ (i = 1, 2, 3, …, n, i is the ith pressure-preserving imaging monitoring). The wave velocity field *V* is the set of wave velocities V (x, y, z) of all discrete microelements of the rock, and x, y, and z are the spatial coordinates of one discrete microelement, which can be found in the literature.

### Damage calculation method

To reduce interference and accurately capture rock failure characteristics, uniaxial multistage loading was employed. Short-term pressure-holding was done at every 50 kN increase in load to minimize acoustic emission interference. This paper does not cover the theory of imaging due to space limitations. For more information, please refer to previous literature^34,35^.

The wave velocity field during loading is denoted as *V*_*i*_ (i = 1, 2, 3, …, n, i is the ith pressure preserving imaging monitoring), and the initial wave velocity field before loading is denoted as *V*_*0*_. The wave velocity field *V*, represents the wave velocities *V* (x, y, z) for all discrete microelements of the rock, where x, y and z are the spatial coordinates of one discrete microelement.

The variable reflecting internal damage in rocks is termed the rock's damage variable. In the realm of rock damage mechanics, the prevailing approach is continuum damage mechanics, necessitating the initial definition of appropriate damage variables. These variables, including elastic constants, yield stress, tensile strength, elongation, density, resistance, ultrasonic wave velocity, and acoustic emission, among others, are defined diversely. Presently, three main types of damage variables are commonly employed in rock mechanics: damage scalar defined by void area, damage tensor defined by void configuration, and damage tensor defined by changes in elastic modulus. Kawamoto et al. presumed rocks to be isotropic bodies composed of rock matrix and microcracks, defining the damage variable using ultrasonic wave velocity^37^.

3 $$D=1-{\left(\frac{{V}_{p}}{{V}_{pf}}\right)}^{2}$$

where ${V}_{p}$ is the acoustic wave velocity (m/s) of isotropic microcracked rock; ${V}_{pf}$ is the acoustic wave velocity (m/s) of intact rock matrix; *D* is the damage variable of the rock.

In this study, the damage variable of each microelement is defined as *D*(*x*, *y*, *z*), and the corresponding wave velocity is defined as* V*(*x*, *y*, *z*). The wave velocity of the microelements before loading is defined as the maximum value of the unladen rock imaging wave velocity field, max(*V*_0_). Damage calculation for each microelement as follow:

4 $$D(x,y,z)=1-(V(x,y,z)/\mathit{max}({V}_{0}){)}^{2}$$

**Figure 3:**
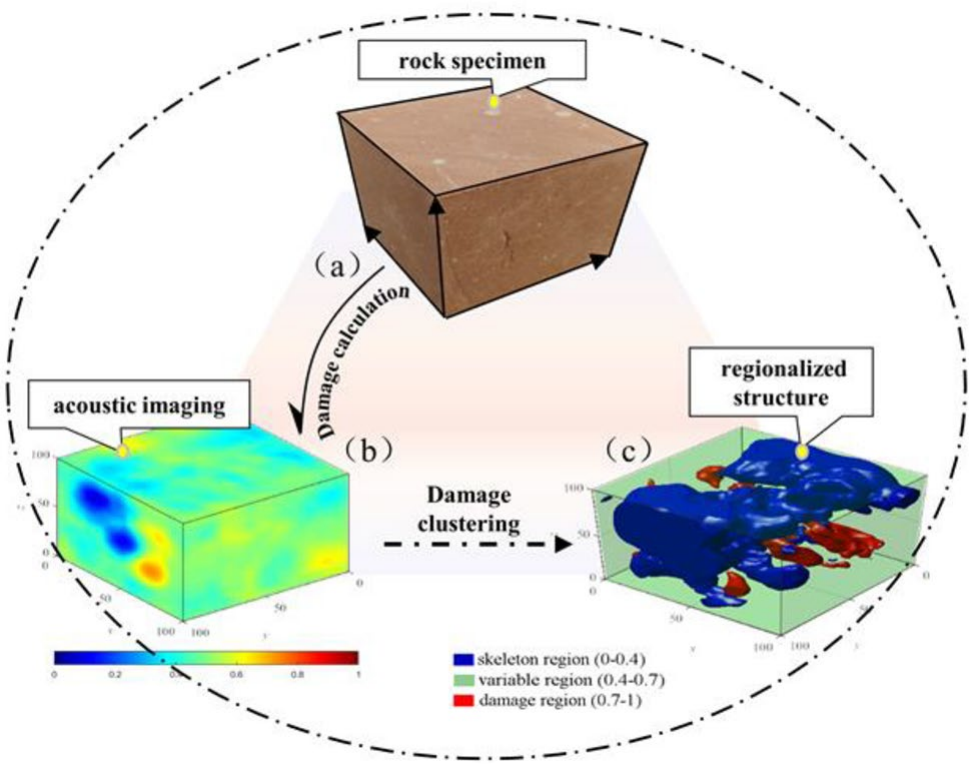
Schematic diagram of rock clustering process. (**a**) The rock specimen (**b**) Damage calculation results for acoustic imaging (**c**) Damage clustering results and regionalization structure.

Damage calculation results for acoustic imaging are shown in Fig. 3.

### Regionalized structure calculation method

Taking one rock specimen as an example (as shown in Fig. 3a) and the corresponding damage calculation result shown in Fig. 3b, the damage analysis exhibits regionalized characteristics within the rock, affecting its structure. However, discrete damage data makes it difficult to reveal underlying patterns. To address this, a K-means clustering analysis was performed based on the damage state in order to explore its overall impact. The element points are randomly selected as the initial center, and the Euclidean distance between the damaged elements and the center damage under different loads is used as the similarity criterion for calculating the clustering process, and the average of the similarity within each cluster is used as the new center damage point. The clustering centers are updated by continuously adjusting the iterations, and the calculations are stopped when the maximum number of iterations is reached. It is useful for grouping objects with similar characteristics into the same cluster. The final iterations result in Fig. 3c, which shows three main types of regionalized structures (Table 1 displays corresponding damage value ranges).

**Table 1:** K-mean clustering results and damage area division.

Project	1	2	3
Clustering center	0.274	0.519	0.819
Minimum wave velocity/(mm/s)	3.07	2.28	0
Maximum wave velocity/(mm/s)	3.96	3.07	2.28
Damage value range	0–0.4	0.4–0.7	0.7–1

**Figure 4:**
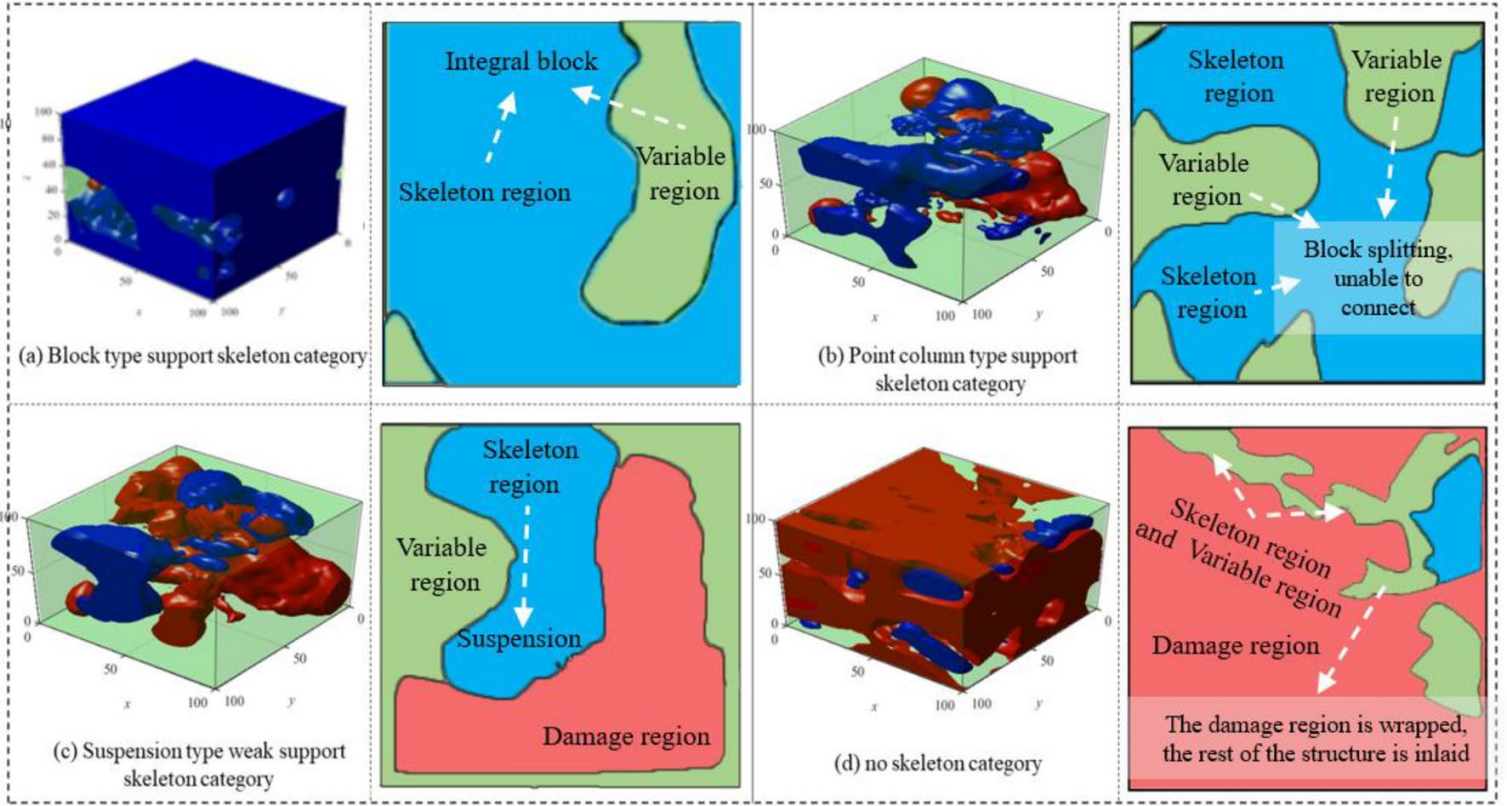
Schematic diagram of spatial distribution of regionalized structures. (**a**) Block-type support skeleton category (**b**) Point-column type support skeleton category (**c**) Suspension-type weak support skeleton category (**d**) No skeleton category.

Statistical analysis of multiple test sets reveals a common objective phenomenon (as shown in Fig. 4). The figure shows a regionalized structure within the rocks, with the blue structure representing lower damage and higher mechanical properties (Compressive strength and Shear strength), the red structure representing higher damage and lower mechanical properties, and the translucent green areas representing intermediate properties. These three structures intertwine to form a complex spatial structure. Based on the clustering results and the role of the structure, we define three regions:

1. Skeleton region: The intact undamaged region with a damage value range of 0–0.4 acts as a skeletal support, providing high intactness and resistance to deformation.
2. Variable region: Region with partial defects (damage value range 0.4–0.7), acts as auxiliary support and a transition to the damage region.
3. Damage region: Region with severe defects (damage value range 0.7–1.0), experiences further degradation and limited ability to transition to skeleton or variable regions.

## Results

### Regionalized structures with different distribution characteristics

Statistical analysis of rock imaging reveals that rocks exhibit complex spatial distribution relationships comprised of three types of regionalized structures. These structures can form various spatial distribution patterns, as depicted in Fig. 4, which highlights the distribution characteristics found in rocks.

1. “Type I rock”: The skeleton and variable regions are relatively complete, forming a supporting structure with a nearly intact room block. The variable or damage region attaches as mosaics without splitting the skeleton region. We defined it as “block-type support skeleton category (as shown in Fig. 4a).”
2. “Type II rock”: The skeleton and variable regions form an irregular columnar support structure against deformation in the direction of maximum principal stress. In the vertical direction of maximum principal stress, the skeleton region is segmented by the variable or damage region without interconnection. We refer to this distribution as the “point-column support skeleton category (as shown in Fig. 4b).”
3. “Type III rock”: The skeleton region does not provide connected support in the maximum principal stress direction. It attaches to the variable region within the rock, forming a suspension-type weak support structure. We classify this distribution as the “suspension-type weak support skeleton category (as shown in Fig. 4c).”
4. “Type IV rock”: The damage region functions as the main support structure in the rock, with sporadic embedding of the skeleton region and variable region. This rock distribution lacks clear regional characteristics and is classified as the “no skeleton category (as shown in Fig. 4d).”

### Rock fracture results of different structural patterns

**Figure 5:**
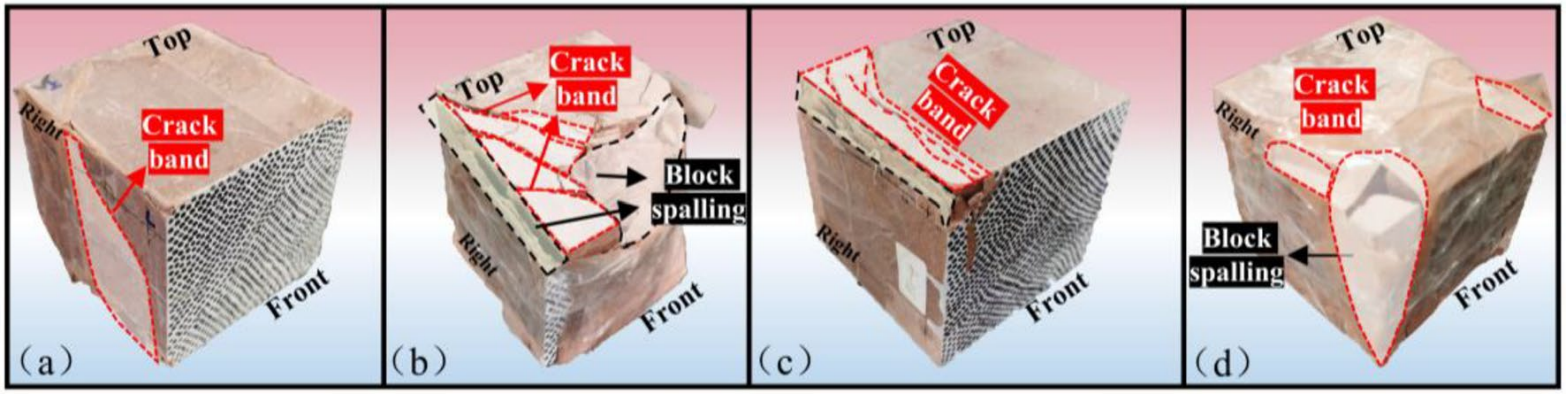
Final failure of four types of rocks. (**a**) Type I rock (**b**) Type II rock (**c**) Type III rock (**d**) Type IV rock.

Rock fracture results of different regionalized structures were analyzed in the current study. A comparison of failure outcomes in four types of rocks revealed distinct differences in their final failure modes. Figure 5 illustrates the final failure situations of these four rock types.

- In type I rock, only a few crack bands intersect as blocks on the surface, leading to a single and less pronounced failure mode.
- Type II rock exhibits a complex failure mode where multiple crack bands intertwine and local rock spalling occurs as blocks.
- Type III rock displays orderly located crack bands in a specific area, with vertical penetration of the rock in the loading direction.
- Type IV rock experiences multiple crack bands that directly traverse the rock, resulting in extensive areas of rock block spalling and a severe failure mode.

### Synergistic evolutionary characteristics of regionalized structures on different rock types

**Figure 6:**
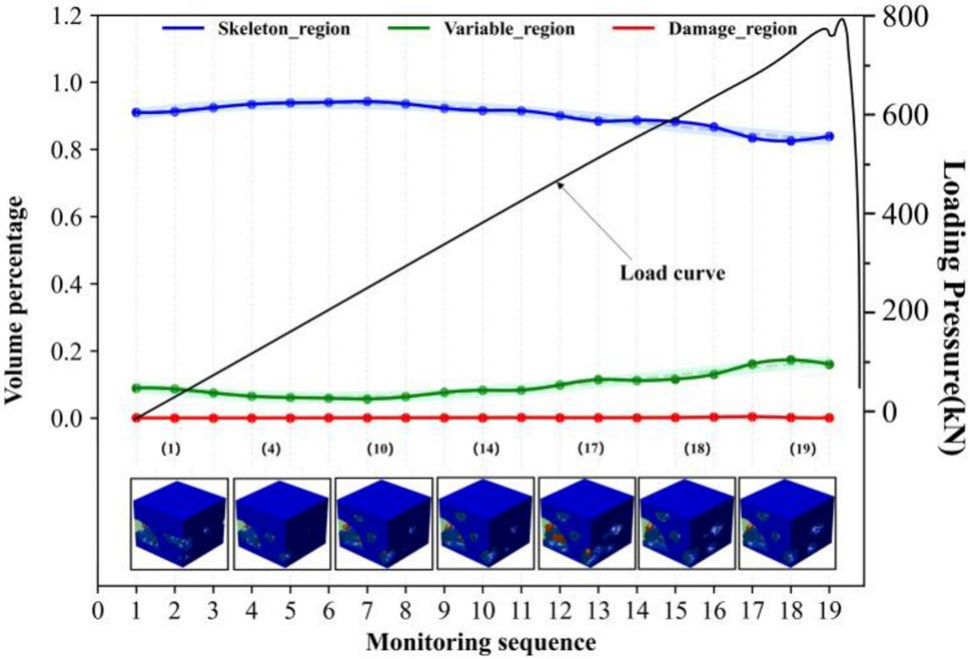
Evolutionary of regionalized structure characteristics in type I rock.

**Figure 7:**
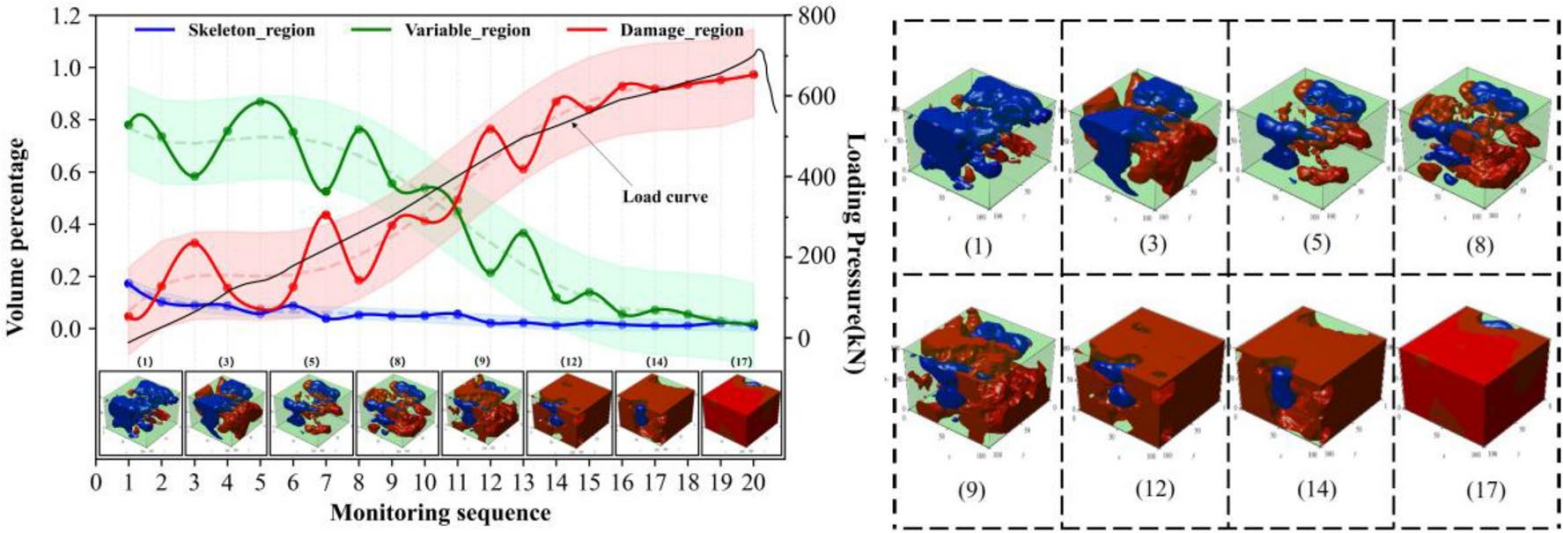
Evolutionary of regionalized structure characteristics in type II rock.

**Figure 8:**
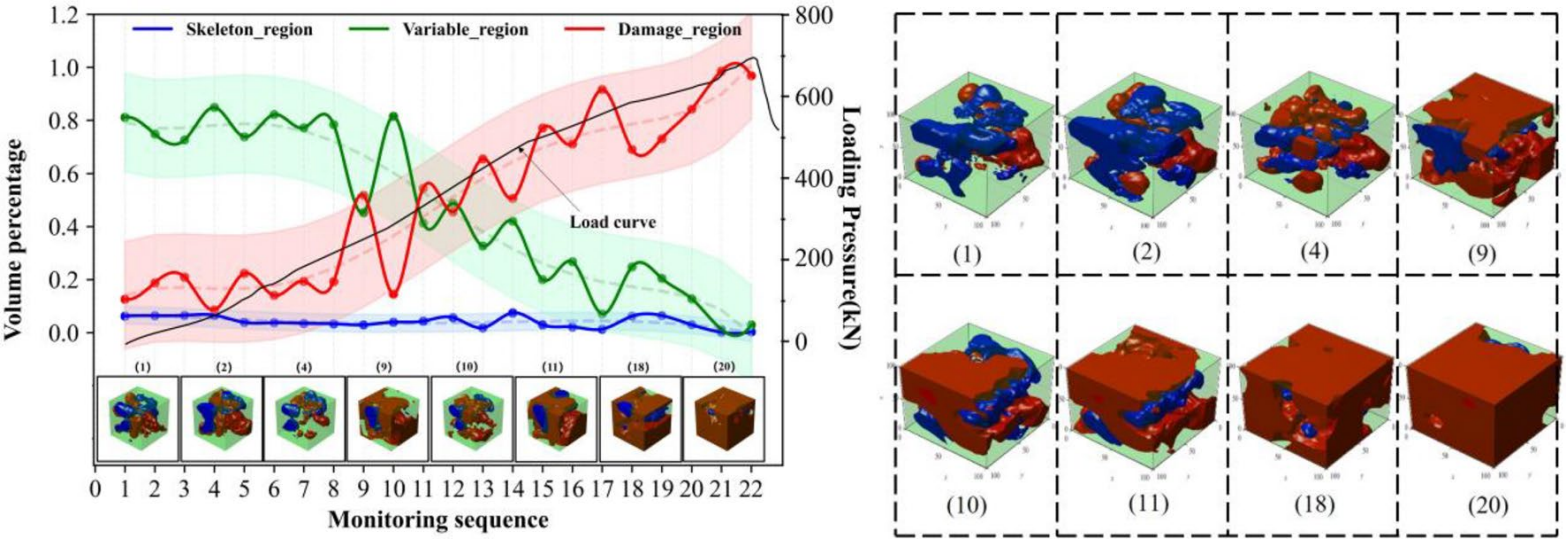
Evolutionary of regionalized structure characteristics in type III rock.

**Figure 9:**
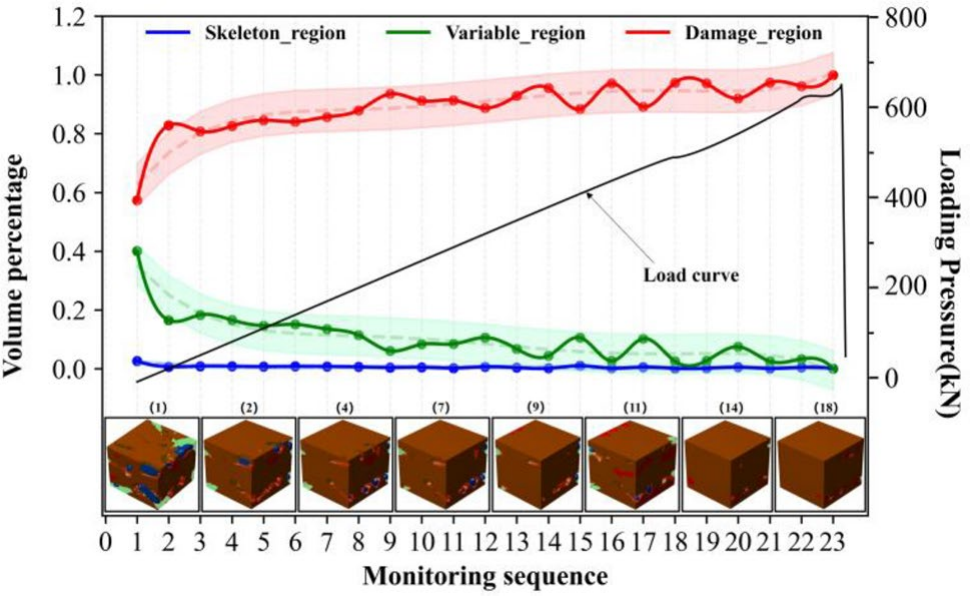
Evolutionary of regionalized structure characteristics in type IV rock.

The observed differences in rock failure can be attributed to the synergistic evolution of regionalized structures influencing the failure process. Further investigations are required to establish the relationships between regionalized structures and failure. Figures 6, 7, 8 and 9 illustrate the synergistic evolutionary characteristics and final failure outcomes of these regionalized structures with different distribution relationships during the failure process.

1. Evolutionary characteristics of regionalized structure in Type I rock.
  In this distribution relationship of regionalized structures, the skeletal region and variable region exhibit high integrity, with the supporting structure forming a nearly complete block-like support. The volume percentage of the skeletal region is relatively high, while the damage region has the lowest volume percentage.
  From the volume proportion changes in Fig. 6, it can be observed that the overall trend of volume proportion changes in the skeleton region, variable region, and damage region is relatively slow during the mechanical loading process. The skeleton region shows a trend of initial increase followed by a decrease, while the variable region exhibits an initial decrease followed by a gradual increase, with small variations, until the rock suddenly fractures. The three-dimensional contour graphs of the skeleton region, variable region, and damage region in Fig. 6 also demonstrate the transformation between them, primarily occurring between the skeleton region and the variable region.
  In type I rocks, the skeletal region primarily bears all the external loading forces. Additionally, due to the strong deformation resistance of the skeletal region, there is relatively little cooperation among the skeletal, variable, and damage region throughout the entire loading process of the rock. As a result, the volume proportions of these three regions exhibit minimal changes.
2. Evolutionary characteristics of regionalized structure in type II rock.
  The skeletal region and variable region have low integrity in this distribution relationship of regionalized structure, and there is the supporting structure of multiple irregular point columns within rocks, resulting in the local supporting structure area being weaker. The volume percentages of the variable region are highest, and the volume percentages of the skeletal region and damage region are lower and relatively closer (as shown in Fig. 7).
  In the early stages of loading, the percentages of the skeletal and variable regions gradually decrease and degrade into the damage region, which leads to the percentage of the damage region increasing. It can be observed from the evolutionary situation that after the type I rocks pass through the evolutionary stage, the subsequent evolution is basically consistent with the evolution of type II and type III rocks.
  Analyzing the synergistic evolutionary mechanism of regionalized structures during loading, in the initial state, the skeletal region is divided into several parts by the variable region in type II rock, and integrity is reduced, which leads to the resistance ability being weakened in this structural distribution. The skeletal region and adjacent regionalized structure have different deformation capacities, which further leads to a difference in those areas with the action of external forces. With the gradual increase of external load, the regionalized structures will gradually deteriorate and evolve sharply between junctions, resulting in the skeletal region evolving into the variable region. The variable region assists the skeletal region in carrying external forces together. Because the resistance capacities of the variable region are weaker, which leads to increasing damage and evolves into the damage region. Finally, the damage regions connect each other and wrap around the other regionalized structures.
  During the loading process of the rock, there is a phenomenon of mutual transformation among the three regions, with the transformation between the variable region and the damage region being particularly prominent. As shown by monitoring points 6–7, 8–9, 12–13, etc., in Fig. 7, the volume proportion of the damage region significantly decreases, with the red damage area in the upper right corner transforming into the light green variable region. This is because the damage region undergoes compaction under stress, leading to partial healing of the damage. Meanwhile, a small amount of the variable region also transforms into the higher-strength skeletal region. From the perspective of the entire rock damage evolution process, there is continuous transformation and alternation among different regions. This indicates that under mechanical loading, internal damage and compaction processes in the rock persist in local areas. This phenomenon is also observed in type III rock and type IV rock (Figs. 8, 9).
3. Evolutionary characteristics of regionalized structure in type III rock.
  The skeletal region exists as suspensions within the rock in this distribution relationship of regionalized structure. The evolution trends of volume percentage of regionalized structures are shown in Fig. 8. The evolution trends of type III rocks and type II rocks are more similar, but there are still differences. The evolution of the skeletal region of type III rocks is more stable than that of type II rocks during loading. In these two types of rocks, the skeletal region is the support structure to resist the external force. Therefore, in terms of the quantitative relationships, the skeletal region is damaged, which causes the volume percentage to express a decreasing trend. Moreover, the volume percentage of the skeletal region in type III rock is slightly less than that in type II rock. It also limits the scope of its variation. In terms of distribution characteristics, the skeletal region exists as suspensions within type III rocks, and the supporting structure is weaker. And the point-column type is inside the type II rocks, at the early stage of stress, the point-column is damaged under the external force, which results in the change to suspension of the skeletal region. Therefore, in the type II rock, there is a local evolution phenomenon from type II to type III rocks within the type II rocks in the initial stage of stress, resulting in a type II and type III composite. The evolution of the local within the rocks is shown in Fig. 8. The skeletal region is distributed at the right and top of the rocks, and the morphology is an integrity column. With the external load, the columns of the skeletal region gradually evolved into suspensions and were wrapped. Therefore, comparing type I rocks with type II rocks, the evolution of the skeletal region of type III rocks is stable during the loading.
  Analyzing the synergistic evolutionary mechanism of regionalized structures during loading, in the initial state of type III rock, due to the skeleton region acting as suspension attached to the variable region, the support is unstable. With the gradual increase in external load, the variable regions mainly carry the load with weak resistance capacities, resulting in increasing internal damage and evolving into the damage region until rock failure.
4. Evolutionary characteristics of regionalized structure in type IV rock.
  The initial damage is extremely serious within type IV rock. In the initial state, there are a large number of damage regions and extremely few skeletal regions and variable regions. In this rock type, the evolution is relatively simple: only the variable region evolves into the damage region in the initial stage.
  Due to these factors, rock damage has been serious, and the damage region’s mechanical properties are weaker than those of the skeletal region and variable region, which makes reverse evolution difficult. This rock type exists less frequently, and the internal damage is more random, so analysis is difficult.

## Discussion

In this study, we made a preliminary discovery regarding the rock’s regionalized structures and their mechanical properties. Specifically, we identified three distinct structures: the “skeletal region,” “variable region,” and “damage region.” Based on the spatial combinations of these structures, we categorized the rock into four types of support skeletons: block type, point-column type, suspension type (weak support), and no skeleton.

When comparing the failure results of the different rock types, we noticed the following:

1. There is a synergistic evolutionary relationship among the regionalized structural bodies during loading, which significantly impacts the failure outcomes.
2. The synergistic evolutionary relationship of the structural bodies differs across the various rock types. Type I rock has a unique stage of evolution and has the potential to transform into other rock types. Type II rock gradually transitions from column groups to suspensions similar to Type III rock, but only in localized areas. On the other hand, Type IV rock experiences severe internal damage, resulting in random distribution of damage within the rock structure.

### Commonalities and differential of evolutionary relationships of regionalized structure

The regionalized structures in the four rock types exhibit a synergistic evolutionary relationship. This is evident in their spatial distribution, with key evolutionary locations concentrated in the junction areas of different structures. Over time, i.e., a gradual increase in uniaxial stress, these structures undergo a similar transformation from skeletal and variable regions to the damage region, gradually encasing other structures within the rock specimen.

**Figure 10:**
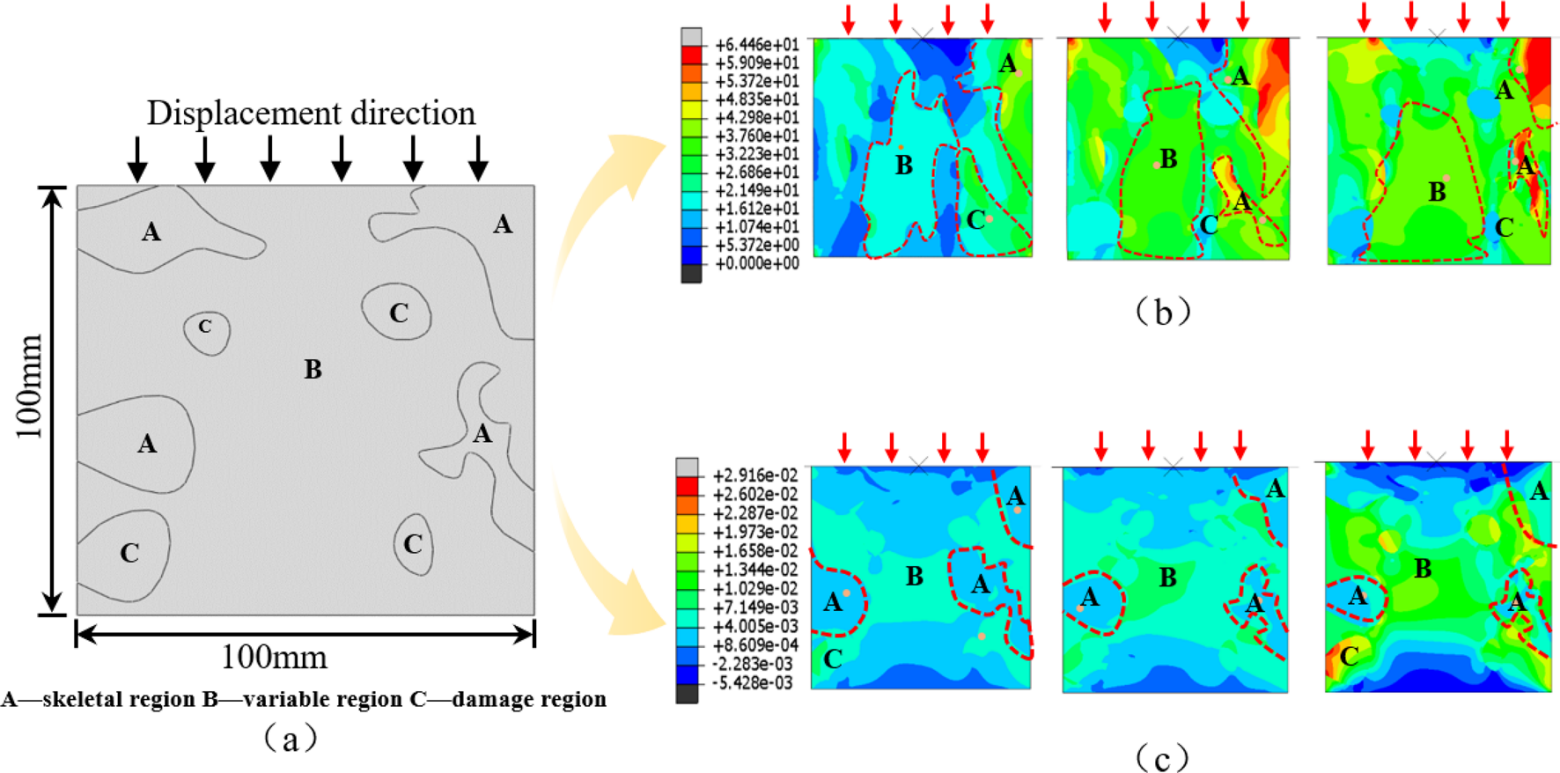
Stress and strain distribution of different structures during rock fracture. (**a**) Regionalized structure distribution in numerical models (**b**) Stress distribution in type II rocks (**c**) Stain distribution in type II rocks.

This synergistic evolution is primarily driven by the varying mechanical properties of adjacent structures. Differences in these properties result in stress disparities at the junctions, which intensify with increasing external load. As regionalized structures reach their bearing capacity, they evolve to the damage region, unable to reverse due to their weak mechanical properties. This leads to an increasing presence of damaged regions until the rock ultimately fails. In order to show the stress disparities between different regionalized structures under uniaxial compression, a finite element numerical simulation analysis was performed to simulate them (as shown in Fig. 10). The areas represent regionalized structures with different mechanical properties, and the results clearly show that the stress difference at the junction of adjacent regionalized structures becomes obvious under stress loading and that there are clear deformation asynchronies in the two areas’ neighboring sections.

However, there are notable differences in the synergistic evolutionary relationship among the four rock types. Type I rock experiences a distinct evolutionary stage, characterized by a complete distribution of the skeletal region internally, providing robust support against external forces. This leads to inevitable deterioration and local transformation of the skeletal region into variable or damage regions, significantly reducing its integrity.

In Type II and Type III rocks, point-column groups or suspensions are present with sporadic supporting structures. This intensifies evolution at the junctions of regionalized structures, resulting in increasing damage regions that gradually encompass the skeletal and variable regions.

Type IV rock exhibits numerous damage regions with weak mechanical properties, making evolution challenging. Consequently, a different synergistic evolutionary relationship is observed, with implications for the final failure of the rock.

In summary, the analysis reveals distinct synergistic evolutionary relationships within rocks, which influence their ultimate failure modes.

### Mechanisms of the evolutionary relationships on structure

To enhance clarity, conciseness, and readability of the analysis, a finite-element numerical simulation was conducted to validate the explanations provided. The square model has dimensions of 100 mm in length, 100 mm in width (As shown in Fig. 10a). The in-built elastoplastic constitutive model was used, and the Mohr–Coulomb criterion of the software was adopted, assuming that each material is isotropic. The displacement constraints were applied to the bottom surface interfaces of the mode, and the boundary conditions of displacement were applied to the top of the model. The model was split into different regions and assigned different damage attributes in order to represent the different regionalized structures according to the actual acoustic wave imaging condition (take the internal slices of type I rocks as an example.). The stress (the direction is the same as the external load, in MPa) and strain distributions of different regionalized structures during the loading process are obtained, and the results are shown in Fig. 10. The results, illustrated in Fig. 10, support the notion that regionalized structures within rocks, characterized by varying mechanical properties, evolve synergistically.

Stress distributions (Fig. 10b) indicate that the principal stresses concentrate in the skeletal region at the top of the rock (area A), suggesting its primary role as the initial support structure. With increasing external load, stress differences become apparent at the junctions of adjacent regionalized structures, focusing stress on the variable region. This region then serves as an auxiliary support structure to assist the skeletal region. As stresses approach the bearing limit of each regionalized structure, stress redistribution occurs, ultimately leading to rock failure.

The deformation capacities of regionalized structural bodies also vary, as evident from the strain distributions (Fig. 10c). The skeletal region undergoes minimal deformation due to its strong mechanical properties and low compressibility. In contrast, other regions experience greater deformation due to their weaker mechanical properties and higher compressibility. As the external load increases, deformation asynchronies occur among adjacent regionalized structures, which is observed in area A and other nearby structures. This supports the notion that key synergistic evolutionary locations primarily exist at the junctions of different types of regionalized structures.

In conclusion, the study highlights the influence of mechanical property differences and the roles played by regionalized structures on their synergistic evolutionary relationships. Failure of the rock typically occurs at the junction areas where different types of regionalized structures meet.

The described relationship between regionalized structure and synergistic evolutionary processes in rocks has important implications for rock mechanics theory and rock engineering. Currently, researchers agree that rock failure involves the accumulation, expansion, and penetration of microstructural damage within the rock. We think that rock is a natural aggregate of minerals formed by combining one or more rock-forming minerals in a specific way, and the isolated mineral grains and the micro-defects are not enough to affect the macro-properties and the rock fracture mode but rather impact the macroscopic properties and fracture model of rock in the form of regionalized assemblages. This study examines the macroscopic perspective of how differences in mechanical properties between regionalized structures within rocks contribute to this process. The changing synergistic evolutionary relationships between regionalized structures, under the influence of external forces, have a direct impact on rock failure. This provides a novel perspective on the mechanism of rock failure. Meanwhile, we will propose the concept of a rock structure system in order to build a bridge between microscopic studies of rock fracture and macroscopic studies.

Moreover, regionalization phenomena are commonly observed in various rock engineering scenarios, including natural and manmade rock masses such as mountain slopes, open slopes, and roadways surrounded by rock. We also hope the concept of a regionalized structure can adapt to the engineering site. In that way, understanding the synergistic evolutionary relationship between regionalized structural elements is crucial for identifying potentially hazardous areas within rock masses. Such understanding plays a vital role in predicting and preventing disasters.

## Conclusions

In conclusion, this study aimed to investigate the synergistic evolutionary relationship between regionalized structures in rocks during failure. It utilized a multistage loading test with acoustic emission-acoustic imaging monitoring technology to analyze the imaging results and propose a definition of different regionalized structures. The research findings can be summarized as follows:

1. The study identified three types of regionalized structures in rocks, namely the skeletal region, variable region, and damage region. Based on the spatial combination relationships within rocks, the study further classified these structures into four complex rock structure types: block-type support skeleton, point-column type support skeleton, suspension type weak support skeleton, and no-skeleton category.
2. The study found both commonalities and differences in the synergistic evolutionary relationship among the four rock types. The adjacent parts of different regionalized structures in rocks become the evolutionary locations due to their varying mchanical properties. The overall evolutionary process is similar, where the variable region becomes dominant, while the skeleton region and variable region gradually decrease and the damage region increases. The block-type support skeleton category exhibits a unique evolutionary stage, while the rocks of the point-column type support skeleton and suspension type weak support skeleton categories show more violent evolution. The rocks of the no skeleton category experience random failure due to weak mechanical properties.
3. The study observed considerable stress differences and asynchronous deformations at the junction locations of different regionalized structural bodies. This phenomenon is caused by the varying mechanical properties and roles of regionalized structures. The change in the synergistic evolutionary relationship between these structures directly affects the final failure of the rock.

While this study provides valuable insights into the synergistic evolutionary relationships of regionalized structures during rock failure, it is important to note that rock failure is a complex phenomenon involving multiple systems. Further research is required to delve deeper into the internal relationships between regionalized structures and rock failure.

## Data Availability

The datasets used and/or analyzed during the current study are available from the corresponding author upon reasonable request.
